# Remote delivery of CBT training, clinical supervision and services: in times of crisis or business as usual

**DOI:** 10.1017/S1754470X20000343

**Published:** 2020-08-03

**Authors:** Paul Cromarty, Dominic Gallagher, Julianne Watson

**Affiliations:** CBT Institute, Adelaide, Australia

**Keywords:** clinical supervision, cognitive behaviour therapy (CBT), COVID-19, Improving Access to Psychological Therapies (IAPT), remote delivery, training

## Abstract

The worldwide coronavirus pandemic has forced health services to adapt their delivery to protect the health of all concerned, and avoid service users facing severe disruption. Improving Access to Psychological Therapies (IAPT) services in particular are having to explore remote working methods to continue functioning. Australian IAPT services have utilised remote delivery methods and disruptive technologies at their core from inception. This was to maintain fidelity and clinical governance across vast distances but has allowed training, supervision and service delivery to continue virtually uninterrupted through coronavirus restrictions. On this basis, key recommendations for remote working are outlined. Remote methods are defined as (1) real time delivery, (2) independent delivery and (3) blended delivery. These are applied across three broad areas of remote training, remote clinical supervision and remote service delivery. Recommendations may be of great benefit to IAPT training institutions, clinical supervisors and service providers considering a move towards remote delivery. Challenges, adaptations and examples of applying remote methods are outlined, including case examples of methods applied to low-intensity and high-intensity cognitive behavioural therapy. Remote methods can safeguard service continuity in times of worldwide crisis and can contribute to reducing the impact of increased mental health presentations post-COVID-19.

**Key learning aims:**

1. To understand the core areas of remote training, clinical supervision and service delivery.
2. To review and distinguish between three broad methods of remote working.
3. To understand how to plan remote working via key recommendations and case examples.

## Introduction

An Australian version of IAPT (Clark, 2011) was piloted in three sites over a 3-year period from 2013 with NewAccess. This brand of IAPT was developed by the charitable organisation Beyond Blue as a low-intensity service. The challenges and success of using an NHS-based model in the semi-private Australian healthcare system has been previously reported (Cromarty *et al*., 2016). These include remote delivery of training and supervision, training of non-graduates and high clinical recovery rates of almost 70%. An independent evaluation reported positive outcomes in retention of low-intensity workers and their satisfaction with training (Beyond Blue NewAccess Demonstration Independent Evaluation, 2015).

In 2015 the Australian Government Review of Mental Health Programmes and Services (Australian Government, 2015) specifically highlighted NewAccess and IAPT, stating, ‘*Primary Health Networks (PHNs) will … be encouraged and supported to work towards better utilisation of low intensity “coaching” services for people with lesser needs, building on evaluations of programmes such as the NewAccess model of care, and the Improving Access to Psychological Therapies model of stepped care implemented in the United Kingdom.*’ A cognitive behavioural therapy (CBT) training team CBT Institute was set up by clinicians that delivered the pilot to continue roll out of NewAccess and similar IAPT initiatives on a national scale. By 2020 a further 16 low-intensity IAPT sites emerged across Australia with utilisation of remote training, supervision and service delivery at their core. In 2017 the first Australian high-intensity IAPT service, Next Step, was commissioned by the PHN for the Australian Capital Territory. Next Step was designed to run alongside the existing low-intensity CBT service in Canberra, which was an original Beyond Blue pilot site.

Training of these Australian low and high-intensity services adheres closely to the UK IAPT Curricula, the National Institute for Health and Care Excellence (NICE) guidelines (NICE, 2011) and stepped care mental health principles (Bower and Gilbody, 2005). IAPT aims to meet the challenge faced by CBT services to produce similar positive outcomes to research studies (Shafran *et al*., 2009). Australian IAPT is underpinned by further evidence of best practice from expert observations, reference groups, consensus statements and key books or papers viewed as authoritative by professional and academic bodies. This includes key concepts from Freeston (2008) in developing focused expertise over breadth of experience, and guidance on clinicians working in rigorous but flexible ways under optimal conditions. Additional features are promotion of single strand interventions for low-intensity (Turpin, 2010) and ‘high dose/narrow bandwidth’ interventions (Cromarty, 2016) across high- and low-intensity steps.

In contrast to the above evidence for practice, there is a small but emergent theoretical and empirical base for CBT training and supervision (Bennett-Levy *et al*., 2009) This increasingly enables trainers to draw on an evidence base of training or supervision methods for attainment of CBT competencies. One example is the declarative, procedural and reflective (DPR) model of therapist skill development, based on information processing theory (Bennett-Levy, 2006). This highlights three principal systems of skills and knowledge acquisition, categorised as declarative, procedural and reflective. This DPR model provides the framework that can account for why different training methods are needed for different elements of therapist skill. Reflection is identified as pivotal to therapist skill development. The DPR model is used as a core framework for remote training and supervision in Australian IAPT.

## Why work remotely?

In Australia there are clear practical advantages in terms of flexibility, optimising resources and cost efficiency, with the ability to deliver CBT training, supervision and services over vast distances. There are three different time zones within Australia. One state, Queensland, does not have daylight saving so for part of the year there are effectively four. Australia is roughly 2500 miles long from east to west. This is roughly the same distance from east to west coasts of the USA or a flight from London to Baghdad. Flying is the primary form of transport between most major cities. Travelling from Melbourne in the state of Victoria to the neighbouring city of Adelaide is a distance of 450 miles, requiring an 8-hour drive or 1 hour flight. In rural and remote areas, IAPT workers may be based in health centres 3 to 4 hours drive from their nearest team members. They already use video or telephone conferencing as standard, for referral meetings and other service requirements. Australia does not have the wealth of postgraduate trained and accredited CBT practitioners and supervisors compared with the multi-professional CBT training and minimum standards of the UK (Cromarty, 2016). This means there are not sufficient trained therapists and supervisors in local services to set up IAPT (Clark *et al*., 2009) with the same structure piloted in the UK. For example, a low-intensity CBT practitioner in rural Western Queensland and a supervisor based in Adelaide would require a 3-hour flight plus a further 19-hour drive to meet face to face. Without remote training and supervision, Australian IAPT services would not be able to match the clinical supervision specifications of the UK model (Turpin and Wheeler, 2011). Given these challenges, remote working is essential for the small pool of trainers and supervisors trained to UK requirements. This established system of remotely delivering IAPT training, clinical supervision and services has proven robust for working through the coronavirus pandemic. Other organisations may benefit from these adaptations when traditional methods of operating are disrupted during such challenging times.

## Remote working definitions

Remote working is defined as any training, supervision or direct clinical contact that is not delivered by face-to-face appointment. In remote CBT training, supervision and practice, the content should be the same as their face-to-face equivalents, with their delivery methods adapted. We have divided this broad definition into three categories or methods of remote working.

### Method 1

Conducted in real time, e.g. audio visual or telephone-based appointments replace the face-to-face session.

### Method 2

Independent delivery, that allows recipients flexibility to undertake an intervention in their own time, e.g. a trainee studying online or a client using guided self-help materials.

### Method 3

Blended delivery, a blend of the above methods such as a mix of real-time online workshops, internet-based, pre-recorded workshops and independent study exercises. In direct clinical work an example would be audio visual or telephone-based therapy and homework between sessions using a manual, workbook or diaries.

All methods can be supported by disruptive technology (Bower and Christensen, 1995) and other resources, to varying degrees. An example is remote service delivery augmented by email or postal delivery of printed materials and text message appointment reminders. Intake of referral, consent, service information and measures can be done in advance, via telephone triage or sent by post or email. In recent years self-help materials such as printed booklets and training materials have increasingly shifted to being delivered via the internet. This shift includes studies of internet-based CBT with reduced therapist input (Andersson and Titov, 2014; Fairburn and Patel 2017; Richards *et al*., 2015).

An example of remote training is online workshops supported by a web portal with resources such as workshop slides, videos and study guides that can be accessed independently outside of training sessions. Most UK health services and training institutions already possess the above resources and technology but utility appears to vary widely. Population density and close proximity between training institutions and services may explain reliance on traditional face-to-face delivery.

## What considerations are required for remote working?

### Remote CBT training

There is preliminary evidence of acceptability and effectiveness of internet-based CBT training (Bennett-Levy *et al*., 2012; Cromarty *et al*., 2016).

Delivering CBT training remotely requires webcams, online audio and reasonable internet connection with telephone audio as back up. A headset may improve sound quality. An online training portal, categorised as a virtual learning environment (VLE) can support training delivery (Weller, 2007) and allow secure submission of assignments including clinical competency recordings. An online platform offering screen sharing and audio-visual connection such as GoTo Meeting or Zoom is recommended. These can be used for free, with a subscription cost for additional features such as screen sharing, recording sessions and virtual breakout facilities.

Initial interviews and role-plays for training places conducted jointly with services are done remotely. The trainer can join remotely and on occasion candidates opt to be interviewed via videoconference to avoid travel. A brief CBT role-play demonstrating basic therapeutic competence must be passed at interview. This additionally examines ability to adhere to instructions, openness to reflect on performance and receive feedback. This is in line with evidence correlating such factors with improved clinical outcomes of low-intensity CBT practitioners (Green *et al*., 2014).

Testing the VLE in advance and ensuring service providers purchase or possess the necessary equipment prior to commencement of training is recommended. During online training and supervision, slowing down the pace of discussions and allowing a gap between speakers is advised due to slight online time lag. In group sessions, having sound muted when not speaking, using built-in chat to type messages and screen sharing all assist communication. Between-session exercises and role-plays make up for lack of practice opportunity compared with face-to-face workshops. CBT can learn from other fields to make learning less didactic, and more interactive and collaborative along the lines of adult education, e.g. the wealth of literature on problem-based learning (PBL) approaches in medical education (Barrows, 1986). A VLE allows submission of written exercises or recordings of role-plays between online sessions for trainers to observe.

DPR remote training example

Remote training on developing clinical skills in the use of delivering a rationale at assessment. A declarative online presentation would be delivered but feedback on this would be delayed until completion of a procedural role-play. This can be conducted via Method 1 in real time, using a virtual breakout facility on the online platform or Method 2, independently after the session. The role-play can be conducted with trainees using telephone or videoconference to build familiarity with the technology prior to use with clients. Reflective feedback from trainees is now sought during a plenary session, e.g. immediately after the role-plays or on subsequent days. The DPR model proposes that procedural ‘experiential’ role-plays allow deeper reflections than feedback after the declarative workshop. Feedback includes client and therapist observations. Role-plays are repeated to allow trainees to experience each role.

## Remote clinical supervision

There is a paucity of literature on remote clinical supervision in CBT. There is preliminary evidence for effectiveness and utility of videoconference, telephone and internet-based clinical supervision, in CBT for problem gambling (Oakes *et al*., 2007) and to a greater extent for anxiety and depression (Cromarty *et al*., 2016; Koivu *et al*., 2016).

Remote supervision can adhere to existing supervision models, e.g. the Cakestand (Armstrong and Freeston, 2006) and evidence-based clinical supervision (EBCS) principles (Milne, 2009). For low-intensity CBT a new category of clinical case management supervision is required (Turpin and Wheeler, 2011). This can be delivered separately to or integrated with clinical supervision. Feedback from supervisors and high-intensity therapists in Australian IAPT is that high-intensity practitioners can benefit from aspects of low-intensity CBT such as periodically incorporating case management supervision into standard clinical supervision. This can assist in combating therapeutic drift (Waller, 2009) in IAPT services and ensure treatment fidelity via focused expertise (Freeston, 2008) and adherence to ‘high dose/narrow bandwidth’ interventions (Cromarty, 2016).

Remote clinical and case management supervision in CBT can be audio visual or telephone based. This needs to be conducted via Method 1 in real time. The same platform as training can be used but remote supervision delivered 1–1 allows focus on the individual and their caseload. Supervision is likely to experience fewer connection and bandwidth issues than training which can require multiple online connections. Supervision can be augmented by Method 3, blended delivery via VLE, for submission between sessions of clinical recordings, reading materials, supervision preparation sheets and supervisor written feedback.

Access for supervisors to the services clinical case management system (CCMS) such as Iaptus or PCMIS to conduct and document clinical supervision is a huge benefit. The latter may require customisation to allow supervisors remote access and entry to certain areas. CCMSs allow an advantage in providing rigorous clinical governance and risk management across vast distances. A practical tip is for supervisors and supervisees to use two computer screens with audio visual platform on one and CCMS on the other, if possible.

A further governance function of clinical and case management supervision is safeguarding of practitioners and service users by systematically assessing and monitoring risk. The primary risk within IAPT services is risk to self via attempted suicide. Supervisors having remote access to a CCMS are key factors in clinical governance and suicide prevention (Cromarty *et al*., 2016). Psychiatry guidance from the COVID-19 Suicide Prevention Research Collaboration and suicide risk and prevention during the COVID-19 pandemic published in the Lancet (Gunnell *et al*., 2020) specifically recommends remote service delivery. The guidance states, ‘*Mental health services should develop clear remote assessment and care pathways for people who are suicidal, and staff training to support new ways of working. Helplines will require support to maintain or increase their volunteer workforce, and offer more flexible methods of working. Digital training resources would enable those who have not previously worked with people who are suicidal to take active roles in mental health services and helplines. Evidence-based online interventions and applications should be made available to support people who are suicidal.*’ This promotes early intervention with NICE-recommended treatments such as low- and high-intensity CBT for clinical depression as effective suicide prevention strategies in themselves. To fulfil this important function remotely, a number of clinical procedures can be followed to support practice that supervisors need to ensure operate efficiently. This can include conducting specific IAPT risk assessments, triggered by measures such as the PHQ-9 (Kroenke *et al*., 2001), policies such as same day recording of session notes and access to a duty supervisor via telephone, within office hours. The latter allows support and discussion for any same day step up due to suicide risk that cannot wait until planned supervision.

DPR remote supervision example

For case management supervision requirements, an agenda for supervision can be emailed in advance by the trainee. The supervisor can adhere to this plus access the online CCMS to view progress notes, measures and risk assessments. This is no different from face-to-face supervision but the role of the CCSM is more prominent in remote supervision. A supervision learning contract for the duration of training with agreed ground rules and individualised learning goals can be reviewed periodically. The above can be conducted via telephone but an audio-visual platform and support of a VLE allows uploading of trainees’ audio recordings of sessions. Uploading files securely via VLE is recommended as opposed to streaming live, which can slow recordings due to variations in internet connection or strain on servers at particular times. The latter can be applied to low and high-intensity CBT supervision.

For clinical supervision requirements, focus on trainee goals for developing competencies can be tailored to specific techniques, disorders or presentations. This can regard application of idiosyncratic case formulation or aspects of a specific CBT model such as delivering treatment for panic disorder or behavioural activation for depression. A declarative aspect is evident via case presentation. This can be made more procedural and experiential by conducting role-plays or reviewing audio recordings of sessions. Trainees are asked to prepare samples of specific recordings with a section of a session that they (1) think was delivered well and (2) think they need to improve on. The supervisor can further enhance procedural and reflective skills by adhering to a Socratic approach., e.g. asking a trainee to identify their own thoughts, feelings and what they were trying to achieve at a particular point of a recording, or role-playing with them acting as the client. The reflective element of DPR is further developed by trainees summarising from declarative case presentation and procedural, role-play or recordings. This reflection would include: what they are hearing in supervisor feedback? What are they observing now? What can they take back into their practice? What will they do the same and what will they do differently next therapy session?

## Remote assessment of clinical competencies

Conducting assessment of CBT competencies remotely is equally beneficial due to the challenges of distance or during a pandemic. Competencies can be assessed remotely via Method 1 in real time if the supervisor observes the appointment via telephone or audio-visual link. This is less intrusive than sitting in the actual room and can be conducted during both face-to-face or remote clinical sessions. A training portal (VLE) allows this to be assessed more flexibly via Method 2, independent delivery, which enable trainees to securely submit audio or visual recordings of clinical sessions to be marked at a convenient time for the clinical supervisor. A validated competency tool, such as the Assessment of Core CBT Skills (ACCS) (Muse *et al*., 2017) can assist this process.

## Remote service delivery

There is preliminary evidence that internet-based CBT (Mullin *et al*., 2015; Richards *et al*., 2015) and a combination of video or telephone-based CBT (Cromarty *et al*., 2016) for anxiety and depression is acceptable to users with no significant difference in clinical outcome compared with face-to-face service delivery.

Remote CBT delivery requires a telephone or computer with webcam, and secure (and in some cases remote) access to a web-based CCMS. A secure, web-based CCMS is valuable to record data, progress notes and basis for receiving clinical supervision and maintaining good practice while protecting confidentiality (Drummond *et al*., 2015).

The standard protocol for low-intensity CBT is one face-to-face assessment followed by five video conference or telephone treatment sessions, 30 minutes each. Service users may opt for face-to-face appointments if convenient or preferred. Certain low-intensity CBT services offer remote intervention only, with video or telephone-based triage, assessment, treatment and follow-up. An example of this is the Australia-wide low-intensity veteran’s health program developed by Beyond Blue for the Australian Defence Force. Low-intensity practitioners are recruited from ex-military personnel and receive referrals for anyone connected to the Australian Defence Force, including family members and civil personnel. These services have to post or email materials such as consent forms, measures, diaries and guided self-help materials. Clinical procedures for step up and risk have to be adapted to conduct assessments and treatments entirely via telephone or video conferencing.

Low-intensity CBT (Bennett-Levy *et al*., 2010) in Australia is already delivered as standard, entirely via telephone/video conference, based on location and user preference, with no adaptation necessary. However, it is unusual for high-intensity CBT to be delivered via digital platforms. Australian high-intensity CBT services offer up to 18 face-to-face appointments as standard. The clinicians are not accustomed to working via these media and may require training, support and socialisation in order to master remote working practices. Due to the COVID-19 pandemic high-intensity CBT services have moved to conducting certain sessions via telephone or video conference. In doing so, high-intensity clinicians can learn a great deal from their low-intensity colleagues who are far more experienced in remote working practices and using technology to conduct clinical work. High-intensity CBT trainees may conduct clinical work face-to-face but they do receive 1 hour per week clinical supervision entirely via telephone or video conference. High-intensity CBT supervision has continued uninterrupted for trainees and qualified therapists during the coronavirus pandemic due to being delivered remotely as standard.

DPR remote service delivery example

Delivering CBT via telephone requires an agreement on appointment time and who will make the call. Posting or emailing low-intensity guided self-help materials, measures and treatment diaries in advance allows joint access in-session. Using telephone requires clinicians to be more aware of auditory cues such as tone of voice, any audible shifts in affect and allowing for silences. Having role-played telephone sessions previously in training based on DPR is of benefit. Clinicians adept at working via telephone ask clients to draw a rationale as they deliver it. They use the analogy of a clockface starting at 12 for a maintenance rationale of triggers, thoughts, feelings and behaviours. This is surprisingly active and collaborative from the outset. Clients with a computer or smartphone can opt for audio-visual appointment and can observe non-verbal cues. Frequent summarising is important by the clinician, as is two-way feedback during remote working. Remote working is predominantly self-directed rather than therapist-assisted, with planning and reviewing graded exposure, behavioural activation and experiments, etc. using diaries and worksheets. In building a case load, regular practice and clinical supervision is a continuation of DPR from training. Client contact repeatedly reinforces the procedural element of DPR, in turn allowing repeated and arguably deeper reflective experiences via weekly clinical supervision.

## Case examples: remote training and supervision

### Specific low-intensity CBT training and supervision case examples

The Australian team delivers low-ntensity CBT training with only ‘Week 1’ of the 12 months training delivered face-to-face. The remainder of the 6-week initial intensive training block is delivered remotely. Following the 6-week block, the trainees build up a caseload of clients and have 90 minutes individual supervision per week and 2 hours group supervision per month, all delivered remotely via videoconference. Client contact after 6 weeks is a continuation of role-play practice as per the DPR model (Bennett-Levy, 2006). In low-intensity CBT, a competency assessment is conducted in weeks 5 and 6 with role-plays of an assessment and a treatment session, which must be passed before seeing live clients. This DPR based competency assessment is an additional level of rigour where practitioners are recruited from non-graduates and non-health professionals, more akin to an apprenticeship model for low-intensity CBT training (Cromarty, 2016). A further 3 days of workshops are delivered remotely via videoconference over the 12 months training. Once qualified, low-intensity CBT practitioners continue to receive 2 hours monthly group supervision and 1 hour of weekly individual supervision.

As a result of repeatedly delivering training and supervision remotely, the Australian trainers have developed expertise in optimising the learning experience via videoconferencing, e.g.

- Providing tutorials in how to use the videoconference platform;
- Socialising trainees/supervisees to ‘videoconference etiquette’ (e.g. muting audio while not speaking, refraining from distracting behaviour, maintaining full attention, etc.);
- Managing interpersonal and group dynamics;
- Conducting role-plays via telephone and video conferencing;
- Observing and marking role-plays and client sessions via telephone and video conferencing.

A version of low-intensity CBT training that is delivered entirely remotely using digital platforms has been developed. This was initially to enable services to replace individual practitioners between training cohorts or recruit new trainees due to expansion. In response to the coronavirus pandemic, this has been adapted further to facilitate totally remote training of whole cohorts of low-intensity CBT practitioners. The first two cohorts trained totally remotely commenced in April 2020. Because Week 1 was traditionally the only part delivered face-to-face, it was the only part to be adapted. It is in Week 1 that the trainees learn to conduct a low-intensity CBT assessment, so much of the week is spent in role-play activities via DPR. Adapting Week 1 from classroom to videoconference required some planning and tweaks to ensure the usual high standards and rigour of CBT training, e.g. utilising an online training platform with virtual breakout facilities. This allows larger groups to divide into smaller sub-pairs or triads for role-plays, etc. without interference from other groups while all being simultaneously accessed and observed by the trainers.

**Figure 1.**
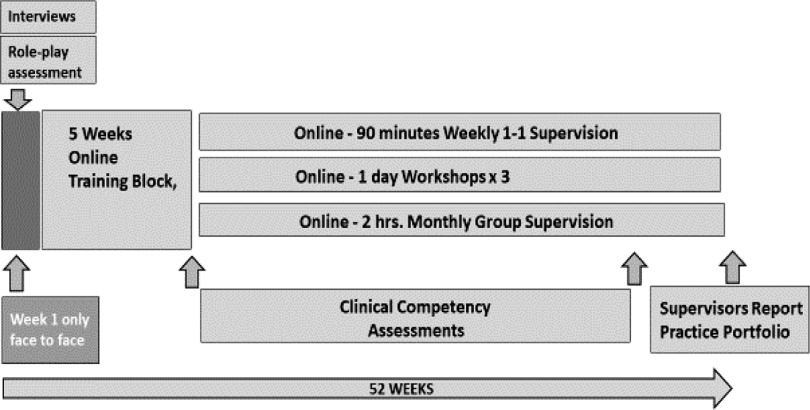
Low-intensity IAPT training timeline.

The DPR model has been adapted to Australian low-intensity CBT settings to provide guidance for trainers and supervisors. For example, the 6 weeks intensive training block heavily emphasises development of procedural skills via repeated role-plays of structured assessments and treatment sessions. This is accompanied by declarative knowledge via teaching on depression and anxiety and treatment protocols such as graded exposure and behavioural activation. Declarative knowledge and reflective skills are developed by repeatedly practising and honing procedural skills. This is implemented in trainee triads, each taking turns to play client, therapist and observer. Client and observer feedback allow the reflective component of DPR. Trainers observe each group in rounds then elicit plenary feedback and overall reflections. Working remotely means that much of the online content is declarative, therefore repeated procedural role-plays are built in during and between sessions to meet skills acquisition requirements. Seeing live clients early in training is a deliberate continuation of the DPR process. This allows trainees to quickly build up a caseload supported by weekly supervision as an ongoing reflective component. This repeated practice over 12 months allows for submission of clinical competencies as a means to assess and endorse CBT skills. See Fig. 1 for a timeline of the low-intensity training.

### Specific high-intensity CBT training and supervision case examples

**Figure 2.**
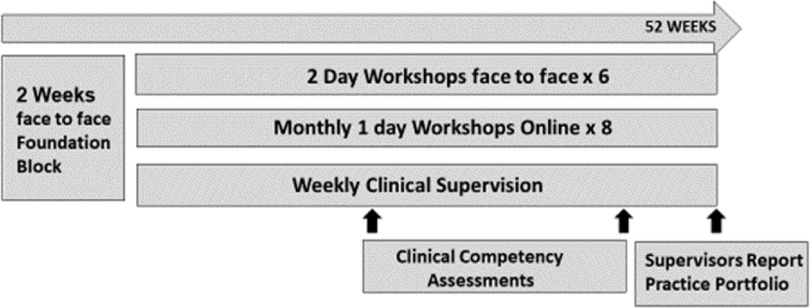
High-intensity IAPT training timeline.

In 2017 Australian high-intensity IAPT training commenced for existing health professionals. The high-intensity service was deliberately designed to run alongside the existing low-intensity service and became Australia’s first stepped care IAPT service with a low-intensity CBT and high-intensity CBT step. High-intensity CBT trainees receive 1 hour per week clinical supervision over the 12 months delivered remotely. On completion of high-intensity CBT training they receive a minimum of 1 hour per month supervision, again delivered remotely. High-intensity trainees see clients and receive CBT supervision after 2-week initial training, with client contact being a continuation of role-play practice as per the DPR model (Bennett-Levy, 2006). High-intensity trainees complete a blend of online and face-to-face workshop in adherence to the IAPT high-intensity curriculum and NICE guidance. This covers more complex presentations than low-intensity CBT including obsessive compulsive disorder and post-traumatic stress disorder. Due to high-intensity trainees already having caseloads, e.g. in their work as psychologists, competency assessments are completed differently from low-intensity training, with a mid- and end-of-course clinical competency. Mid-course competency requires submission of a CBT assessment session. End-of-course competency requires submission of two treatment sessions, one for depression and one for anxiety. These are assessed using a validated competency tool, the ACCS (Muse *et al*., 2017). See Fig. 2 for a timeline of the high-intensity training.

## Summary

Working remotely offers flexibility for service users and case management efficiencies for workers to tackle practical problems of distance, travel and time. Operating remotely is essential across vast distances. It can be adopted by face-to-face services temporarily or partially in times of crisis such as pandemics. Certain resources and requirements are needed to optimise this. Service providers operating remotely have noticed few or no disruptions due to coronavirus even with workers and clients in isolation. The IAPT Competencies Frameworks (ucl.ac.uk/research/research-groups/core/competence-frameworks) for therapy and supervision could expand to include basic competencies for remote working. Interventions and principles for remote training, supervision and therapy are the same as face-to-face CBT, with the difference being in the delivery method. Caution should be expressed during a pandemic with limited opportunities for clients to conduct ‘socially based’ experiments or homework activities. CBT may require more creativity with idiosyncratic treatment plans during periods of enforced isolation. In some cases, this may be worth postponing if sufficient opportunities to test the model cannot be made possible. Delivering low-intensity CBT training and therapy entirely remotely appears an acceptable option to clinicians and service users. Certain high-intensity CBT skills workshops may be better delivered face-to-face. If delivered online, the procedural and reflective components may require augmentation. This can be done in-session using virtual breakout facilities of the VLE for skills role-plays and exercises or by setting them between online sessions. Additionally, clinical supervision can incorporate these more active training and education elements on top of case discussions. A DPR model can facilitate and structure skills acquisition during remote learning. The evidence base for delivering CBT by more flexible methods is emergent but further research is required. Further randomised controlled research into remote service delivery, building on Richards *et al*. (2015) are required, as are more randomised controlled trials to strengthen the evidence for or against remote training and supervision. Any prevailing attitude that working face-to-face is the gold standard in training and service delivery may need to be suspended until additional evidence is gathered.

Given the projected increased prevalence of mental health problems post-coronavirus (Gunnell *et al*., 2020) offering services remotely may prove to be a preventative measure or to at least minimise the scale of impact. There is already a preliminary CBT model based on tolerance of uncertainty distress in the context of coronavirus with further research in progress (Freeston *et al*., 2020). Soon clinicians may be able to incorporate this into remote working practices. There is no evidence to suggest that working remotely correlates with the higher clinical outcomes of Australian IAPT but its role at the core of training, supervision and service delivery certainly signifies that it does not impair clinical recovery rates.

## Key practice points

1. Remote working requires adherence to the same principles of face-to-face CBT training, supervision and practice with adapted delivery methods allowing enhanced resource allocation.
2. Remote working has advantages of flexibility and service continuity due to challenges of time and location and in times of crisis such as pandemics.
3. Remote working requires preparation and resource installation of disruptive technology to varying degrees of reliance.
4. Blending the methods of remote working tailored to idiosyncratic needs of CBT trainers, supervisors and practitioners.
5. Remote clinical and case management supervision has an important clinical governance role in safeguarding and suicide prevention. To optimise best practice for remote working, supervisor access to an online Clinical Case Management System, supported by additional clinical procedures is recommended.

## References

[ref1] Andersson G. , & Titov N. (2014). Advantages and limitations of internet-based interventions for common mental disorders. World Psychiatry, 13, 4–11. 24497236 10.1002/wps.20083 PMC3918007

[ref2] Armstrong, P. V. , & Freeston, M. H. (2006). Conceptualising and formulating cognitive therapy supervision In Case Formulation in Cognitive Behavioural Therapy, ed. Nicholas Tarrier . New York, NY, USA: Routledge/Taylor & Francis Group.

[ref3] Australian Government (2015). Response to Contributing Lives, Thriving Communities – Review of Mental Health Programmes and Services (November 2015), www.health.gov.au

[ref4] Barrows, H. S. (1986). A taxonomy of problem-based learning methods. Medical Education, 20, 481–486. 3796328 10.1111/j.1365-2923.1986.tb01386.x

[ref100] Bennett-Levy, J. (2006). Therapist skills: a cognitive model of their acquisition and refinement. Behavioural and Cognitive Psychotherapy, 34, 57–78.

[ref5] Bennett-Levy, J. , Hawkins, R. , Perry, H. , Cromarty, P. , & Mills, J. (2012). Online cognitive behavioural therapy (CBT) training for therapists: outcomes, acceptability, and impact of support. Australian Psychologist, 47, 174–182.

[ref6] Bennett-Levy, J. , McManus, F. , Westling, E. & Fennell, M. (2009). Acquiring and refining CBT skills and competencies: which training methods are perceived to be most effective? Behavioural and Cognitive Psychotherapy, 37, 571–583 19703329 10.1017/S1352465809990270

[ref7] Bennett-Levy, J. , Richards, D. , Farrand, P. , Christensen, H. , Griffiths, K. , Kavanagh, D. , Klein, B. , Lau, M.A. , Proudfoot, J. , Ritterband, L. White, J. , & Williams, C. (2010). Oxford Guide to Low Intensity CBT Interventions. Oxford University Press

[ref8] Beyond Blue (2015). NewAccess Demonstration Independent Evaluation, https://www.beyondblue.org.au/

[ref9] Bower, J. L. , & Christensen, C. M. (1995). Disruptive technologies: catching the wave. Harvard Business Review, 73, 43–53.

[ref10] Bower, P. , & Gilbody, S. (2005). Stepped care in psychological therapies: access, effectiveness and efficiency narrative literature review. British Journal of Psychiatry, 186, 11–17. 15630118 10.1192/bjp.186.1.11

[ref11] Clark, D. M. (2011) Implementing NICE guidelines for the psychological treatment of depression and anxiety disorders: the IAPT experience. International Review of Psychiatry, 23, 318–327. 22026487 10.3109/09540261.2011.606803 PMC3212920

[ref12] Clark, D. M. , Layard, R. , Smithies, R. , Richards, D. A. , Suckling, R. , Wright, B. (2009). Improving access to psychological therapy: initial evaluation of two UK demonstration sites. Behaviour Research& Therapy, 47, 910–920. 19647230 10.1016/j.brat.2009.07.010 PMC3111658

[ref13] Cromarty, P. (2016). Improving Access to Psychological Therapies (IAPT) in Australia: Evidence-based CBT Interventions for Anxiety, Depression and Gambling Addiction. Innovations and Future Directions in the Behavioural and Cognitive Therapies, ed. R. Menzies, M. Kyrios & N. Kazantis. Australian Academic Press.

[ref14] Cromarty, P. , Drummond, A. , Francis, T. , Watson, J. & Battersby, M. (2016) NewAccess for Depression and Anxiety: Adapting the UK Improving Access to Psychological Therapies Program across Australia: Australasian Psychiatry. 10.1177/1039856216641310 27034440

[ref15] Drummond, A. , Cromarty, P. , & Battersby, M. (2015). Privacy in the digital age: implications for clinical practice. Clinical Psychology: Science and Practice, 22, 227–237.

[ref16] Fairburn, C. G. , & Patel, V. (2017) The impact of digital technology on psychological treatments and their dissemination. Behaviour Research & Therapy, 88, 19–25. doi: 10.1016/j.brat.2016.08.012 28110672 PMC5214969

[ref17] Freeston, M. H (2008). Clinical Art and Clinical Science in CBT: Challenges for Dissemination, Education, Training and Supervision; Keynote at 36th BABCP Annual Conference, Edinburgh, UK.

[ref18] Freeston, M. H. , Tiplady, A. , Mawn, L. , Bottesi, G. , & Thwaites, S. (2020) Towards a model of uncertainty distress in the context of Coronavirus (Covid-19). Personal communication. Under rapid review at psyarxiv.com. 10.1017/S1754470X2000029X PMC7426588 34191941

[ref19] Green, H. , Barkham, M. , Kellett, S. , Saxon, D. (2014). Therapist effects and IAPT psychological wellbeing practitioners (PWPs): a multilevel modelling and mixed methods analysis. Behaviour Research and Therapy, 63, 43–54. 25282626 10.1016/j.brat.2014.08.009

[ref20] Gunnell, D. , Appleby, L. , Arensman, E. , Hawton, K. , John, A. , Kapur, N. , Khan, M. , O’Connor, R. , & Pirkis, J. (2020). COVID-19 suicide risk and prevention during the COVID-19 pandemic. *Lancet Psychiatry*, published online 21 April 2020, 10.1016/S2215-0366(20)30171-1 PMC7173821 32330430

[ref21] Koivu, B. , Drummond, A. , Battersby M. , & Cromarty, P. (2016). Large reductions in depression and anxiety via low intensity CBT delivered by novice coach. Australian and New Zealand Journal of Psychiatry, 50. 10.1177/0004867415624971 26764369

[ref22] Kroenke, K , Spitzer, R. L. & Williams, J. B. (2001). The PHQ-9: validity of a brief depression severity measure. Journal of General Internal Medicine, 16, 606–613. 11556941 10.1046/j.1525-1497.2001.016009606.x PMC1495268

[ref23] Milne, D. (2009). Evidence-Based Clinical Supervision: Principles and Practice. Blackwell Publishing; British Psychological Society.

[ref24] Mullin, A. , Dear, B. F. , Karin, E. , Wootton, B. M. , Staples, L. G. , Johnston, L. , Titov, N. (2015). The UniWellbeing course: a randomised controlled trial of a transdiagnostic internet-delivered cognitive behavioural therapy (CBT) programme for university students with symptoms of anxiety and depression. Internet Interventions, 2, 128–136.

[ref25] Muse, K. , McManus, F. , Rakovshik, S. , & Thwaites, R. (2017). Development and psychometric evaluation of the Assessment of Core CBT Skills (ACCS): an observation-based tool for assessing cognitive behavioral therapy competence. Psychological Assessment, 29, 542–555. 27668487 10.1037/pas0000372

[ref26] NICE (2011). Common mental health disorders: identification and pathways to care. NICE Clinical Guideline 123. Retrieved from: http://www.nice.org.uk/CG123 10.3399/bjgp12X616481 PMC3252532 22520681

[ref27] Oakes, J. , Battersby, M. , Pols, R. , & Cromarty, P. (2007). Exposure therapy for problem gambling via videoconferencing. Journal of Gambling Studies, 24, 107–118. 17846871 10.1007/s10899-007-9074-4

[ref28] Richards, D. , Timulak, L. , O’Brien, E. , Hayes, C. , Vigano, N. , Sharry, J. , & Doherty, G. (2015). A randomized controlled trial of internet-delivered treatment: its potential as a low-intensity community intervention for adults with symptoms of depression. Behaviour Research and Therapy, 75, 20–31. 26523885 10.1016/j.brat.2015.10.005

[ref29] Shafran, R. , Clark, D. M. , Fairburn, C. G. , Arntz, A. , Barlowe, D. H. , Ehlers, A. , Freeston, M. H. , Garety, P. A. , Hollon, S. D. , Ost, L. G. , Salkovskis, P. M. , Williams, J. M. G. , & Wilson, G. T. (2009). Mind the gap: improving the dissemination of CBT. Behaviour Research and Therapy, 47, 902–909. 19664756 10.1016/j.brat.2009.07.003

[ref30] Turpin, G. (2010). Good practice guidance on the use of self-help materials within IAPT services. www.iapt.nhs.uk.

[ref31] Turpin, G. , & Wheeler, S. (2011). IAPT supervision guidance. Retrieved from: http://www.iapt.nhs.uk/silo/files/iapt-supervision-guidance-revised-march-2011.pdf (accessed 18 August 2011).

[ref32] Waller, G. (2009). Evidence-based treatment and therapist drift. Behaviour Research and Therapy, 47, 119–127. 19036354 10.1016/j.brat.2008.10.018

[ref33] Weller, M. (2007). Virtual Learning Environments: Using, Choosing and Developing your VLE. London, UK: Routledge.

